# Dexamethasone Enhanced Functional Recovery after Sciatic Nerve Crush Injury in Rats

**DOI:** 10.1155/2015/627923

**Published:** 2015-03-09

**Authors:** Xinhong Feng, Wei Yuan

**Affiliations:** ^1^Department of Neurology, Beijing Tsinghua Changgung Hospital, Medical Center, Tsinghua University, No. 168 Li Tang Road, Changping District, Beijing 102218, China; ^2^Department of Spine Surgery, Aviation General Hospital of China Medical University, Beijing Institute of Translational Medicine, Chinese Academy of Sciences, No. 3 Anwai Beiyuan Road, Chaoyang District, Beijing 100012, China

## Abstract

Dexamethasone is currently used for the treatment of peripheral nerve injury, but its mechanisms of action are not completely understood. Inflammation/immune response at the site of nerve lesion is known to be an essential trigger of the pathological changes that have a critical impact on nerve repair and regeneration. In this study, we observed the effects of various doses of dexamethasone on the functional recovery after sciatic nerve crush injury in a rat model. Motor functional recovery was monitored by walking track analysis and gastrocnemius muscle mass ratio. The myelinated axon number was counted by morphometric analysis. Rats administered dexamethasone by local intramuscular injection had a higher nerve function index value, increased gastrocnemius muscle mass ratio, reduced Wallerian degeneration severity, and enhanced regenerated myelinated nerve fibers. Immunohistochemical analysis was performed for CD3 expression, which is a marker for T-cell activation, and infiltration in the sciatic nerve. Dexamethasone-injected rats had fewer CD3-positive cells compared to controls. Furthermore, we found increased expression of GAP-43, which is a factor associated with development and plasticity of the nervous system, in rat nerves receiving dexamethasone. These results provide strong evidence that dexamethasone enhances sciatic nerve regeneration and function recovery in a rat model of sciatic nerve injury through immunosuppressive and potential neurotrophic effects.

## 1. Introduction

The specialized structure of a peripheral nerve bundle is essential for normal sensory, motor, and autonomic functions. Injury to peripheral nerves results in temporary or life-long neuronal dysfunction that can subsequently lead to economic or social disability [1]. The present study found that Wallerian degeneration of nerve fibers occurred after peripheral nerve injury. Wallerian degeneration refers to the sequence of events following nerve transection. Similar events that damage axons after a blunt or crush injury are termed Wallerian-like degeneration. Lacerations (transections) as well as stretch and compression (crush) injuries are the most common types of peripheral axon injuries [2]. Traumatic injury to peripheral nerves produces abrupt tissue damage at the lesion site where physical impact occurred. Nerve stumps that are located at distal sites then undergo the cellular changes that characterize Wallerian degeneration, despite not encountering the physical trauma directly. Following an injury, axons break down, Schwann cells reject the myelin portion of their membranes, and bone marrow derived macrophages are recruited and activated together with resident Schwann cells to remove degenerated axons and myelin. There is currently no medical treatment available that can overcome the limitations in axonal regeneration and ensure the recovery of normal sensory and motor functions following nerve trauma. Therefore, new therapeutic intervention strategies for peripheral nerve repair are critically needed. A better understanding of the molecular and cellular mechanisms involved in successful axon regeneration and appropriate target reinnervation would be helpful in developing new therapeutic applications.

Neural-immune interactions are known to occur in response to disease and/or trauma of the nervous and immune system [3]. Immune activation and the subsequent release of immune mediators in the peripheral nervous system have important effects on nerve degeneration and regeneration. A variety of peripheral immune cells, including mast cells, macrophages, and lymphocytes, reside in peripheral nerves and/or are recruited to sites of peripheral nerve injury [4–6]. Secondary injury after peripheral nerve injury caused by the immune response aggravates Wallerian degeneration, which consequently inhibits the repair and regeneration of peripheral nerves [7, 8]. Thus, elucidation of the regulatory components involved in the ensuing inflammatory reaction to neural injury holds great promise in the development of effective treatment strategies to maximize immune elements critical for repair, while simultaneously suppressing aspects of the immune response responsible for further damage to offset disease or injury progression.

Dexamethasone is an anti-inflammatory glucocorticoid that is often used after injury to treat neural inflammation. A previous rat peripheral nerve crush injury study demonstrated synergistically protective effects of topically administered dexamethasone and vitamin B12 on significant improvement in motor function compared to control rats [9]. In addition, Mohammadi et al. showed that after local application of dexamethasone in a transected sciatic nerve rat model, peripheral nerve repair and target organ reinnervation were accelerated [10]. It is especially tempting to associate the immunosuppressant and potential neurotrophic effects induced by dexamethasone with its actions at the site of the nerve injury, which would lead to reduced infiltration of inflammatory cells and production of inflammatory mediators. To the best of our knowledge, this hypothesis has never been explored. Therefore, in the present study we investigated the effects of dexamethasone on functional and histological changes following sciatic nerve crush injury. We applied dexamethasone through local intramuscular injection around the site of nerve injury to reduce side effects of glucocorticoids administered systemically. To identify the mechanism that possibly mediates the effect of dexamethasone on functional recovery after sciatic nerve crush injury, we investigated levels of CD3-positive cells as well as GAP-43 expression.

## 2. Material and Methods 

### 2.1. Animals

A total of 120 adult male Sprague-Dawley rats with an average body weight of 275 g (250–300 g) (Department of Animal Center, Military Academy of Medical Sciences, Beijing, China) were used in this study. The rats were maintained under specific pathogen-free laboratory conditions on a 12 h light/dark cycle with free access to pellet food and water. Rat care and all experimental procedures were in accordance with the Guide for the Care and Use of Laboratory Animals and approved by the Chinese National Committee to the Use of Experimental Animals for Medical Purposes, Beijing Branch.

### 2.2. Grouping and Sciatic Nerve Surgery

The rats were randomly divided into five groups (*n* = 24), anesthetized with an intraperitoneal injection of 10% chloral hydrate (3 mL/kg body weight), and shaved and washed with antiseptic solution before positioning for surgery. The left sciatic nerve was exposed through a gluteal muscle-splitting incision. Surgery was performed on the four experimental groups by clamping the left sciatic nerve for 60 s using pincers with 2 mm width; complete crush was confirmed by the presence of a translucent band across the nerve. The incision was then closed in layers (muscle and skin) with absorbable sutures. In the sham group, the sciatic nerve was exposed but not crushed. After the surgery, the rats were treated as follows: in group (a) (sham group), 0.9% saline was injected into incision site; in group (b) (control group), 0.9% saline was injected into the injured site; in group (c), dexamethasone (0.5 mg/kg) was injected into the injured site; in group (d), dexamethasone (1 mg/kg) was injected into the injured site; and in group (e), dexamethasone (2 mg/kg) was injected into the injured site. The injections were performed once daily for 10 days based on rat weight and clinical protocol. At 7, 14, 21, and 28 days, 6 rats were randomly selected from each group for evaluation of the sciatic functional index (SFI). The rats were then sacrificed and 1 cm of the left sciatic nerve was immediately taken from each rat as an experimental sample.

### 2.3. Sciatic Functional Index

Evaluation of SFI was performed on days 7, 14, 21, and 28 following surgery. Rats were held by the chest and their hind feet were pressed down onto a stamp pad soaked with water-soluble blue ink. Rats were immediately allowed to walk along a confined walkway (7.5 cm wide, 60 cm long) with a dark shelter at the end of the corridor. The walkway was lined with paper on the floor to capture the rat footprints. The following measurements were taken from the footprints: (1) distance from the heel to the third toe, the print length (PL); (2) distance from the first to fifth toe, the toe spread (TS); and (3) distance from the second to the fourth toe, the intermediary toe spread (ITS). All three measurements were taken from the experimental (E, undergoing sciatic nerve crush) and normal (N) limbs. Three factors that comprise the SFI were calculated as follows: (1) print length factor (PLF) = (EPL − NPL)/NPL; (2) toe spread factor (TSF) = (EST − NST)/NST; and (3) intermediary toe spread factor (ITF) = (EIT − NIT)/NIT. Using these data, the SFI, which indicates the differences between the injured and the intact contralateral paw, was calculated by the following formula derived by Bain et al. [11]:

(1) SFI−38.3EPL−NPLNPL+109.5ETS−NTSNTS+13.3EIT−NITNIT−8.8.s.

An SFI equal to −100 indicates significant impairment, whereas an SFI oscillating around 0 is considered to reflect normal function.

### 2.4. Gastrocnemius Muscle Mass Ratio

Recovery assessment was also indexed using the weight ratio of the gastrocnemius muscles 28 days after surgery. Immediately after animals were sacrificed, gastrocnemius muscles were dissected and carefully harvested from both intact and injured sides and weighed while still wet using an electronic balance. All measurements were made by two blinded observers. Values were expressed as a ratio of the wet weight of the gastrocnemius muscle of the operated side to the wet weight of the normal side.

### 2.5. Immunohistochemistry

The left sciatic nerve was dissected, fixed in 4% paraformaldehyde solution for 48 h, conventionally dehydrated, cleared, and embedded in paraffin at a 4 *μ*m thickness. The slice was dewaxed and incubated in 3% H_2_O_2_ at room temperature for 10 min to inactivate endogenous peroxidase. After three washes, the slices were repaired for 10 min in 0.01 M citrate buffer (pH 6.0) with a microwave. After three more washes, primary antibody diluted in PBS with 5% goat serum was added as follows: anti-CD3 (1 : 500, Abcam, USA) and anti-GAP-43 (1 : 200, Epitomics, USA). The following day, the slices were washed with PBS and then secondary antibody labeled with biotin (ZSGB Biotech, Beijing, China) was added. Streptavidin labeled with horseradish peroxidase was added and slices were incubated at room temperature for 30 min. The antigens were visualized with DAB. The sections were washed with double distilled water, air-dried, and fixed with neutral balsam. Sections were visualized on a Leica DM6000B microscope (Leica, Germany) and processed with Image-Pro Plus 6.0 software. T-cell quantification (CD3-labeled cells) was performed on four sections/animal (six animals/group) in the injury region of the crushed sciatic nerves at a position of 5 mm distal to the crush site. GAP-43 protein expression in the sciatic nerve was also measured and processed by this method using the average optical density (mean density). First, the yellow light density sum (IOD SUM) was measured for each photo, and then the measurement area was selected. Lastly, the mean density in the light was calculated using the formula: mean density = (IOD SUM)/area.

### 2.6. Histological Evaluation

On day 28 after surgery, the sedated rats were euthanized and the distal segment of the sciatic nerve was removed and fixed in 10% buffer formal saline. The fixed sciatic nerves were routinely processed for paraffin embedding. Thin sections (4-5 *μ*m) were cut using a microtome and stained with hematoxylin and eosin (H&E) or osmium tetroxide and examined using a light microscope. Myelinated axons were quantified according to a stereological sampling approach as previously described [12].

### 2.7. Statistical Analysis

The experimental data were expressed as means ± SD. The statistical significance of differences between groups was determined by a one-way analysis of variance (ANOVA) followed by Duncan's test for multiple comparisons. A value of *P* < 0.05 was considered statistically significant. Statistical analyses were performed using SPSS version 19.0 (SPSS, Inc., Chicago, IL, USA).

## 3. Results

### 3.1. Motor Functional Recovery

**Figure 1:**
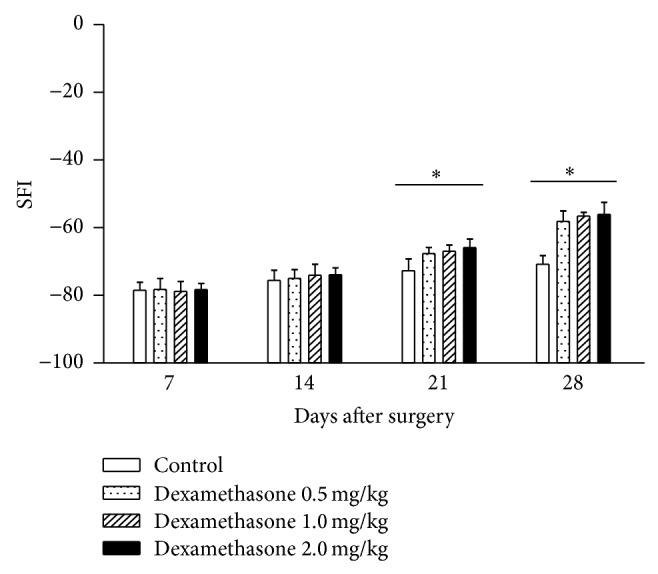
Effects of dexamethasone on the sciatic function index (SFI) 7, 14, 21, and 28 days after surgery. Rats were administered local intramuscular injections with 0.5, 1, or 2 mg/kg dexamethasone, or saline once daily after surgery. The values are represented as means ± SD for 6 rats per group. ^*^Significant difference compared to control, *P* < 0.05.

Figure 1 shows the effects of dexamethasone on SFI on days 7, 14, 21, and 28 after sciatic nerve crush injury. SFI values for the sham group were near zero at all time points (data not shown), indicating normal sciatic nerve function. Seven days after surgery, walking track analysis demonstrated a dramatic decrease of SFI to approximately −78.48 ± 2.4, indicating complete loss of function. At this time point, SFI values showed no significant differences among the various groups. However, 21 days after the injury, SFI values returned to −67.61 ± 2.3, −66.99 ± 1.8, and −65.84 ± 2.5 in groups (c), (d), and (e), respectively, and were significantly higher than group (b) (−72.74 ± 3.5) (*P* < 0.05). No significance was observed among groups (c), (d), and (e) (*P* > 0.05). Furthermore, 28 days after the injury, SFI values returned to −58.15 ± 3.1, −56.56 ± 1.7, and −56.07 ± 3.5 in groups (c), (d), and (e), respectively, and were significantly higher than group (b) (−70.79 ± 2.5) (*P* < 0.05) (Figure 1). These results suggest that dexamethasone promoted the recovery of SFI in the injured sciatic nerves.

### 3.2. Gastrocnemius Muscle Mass Ratio

**Figure 2:**
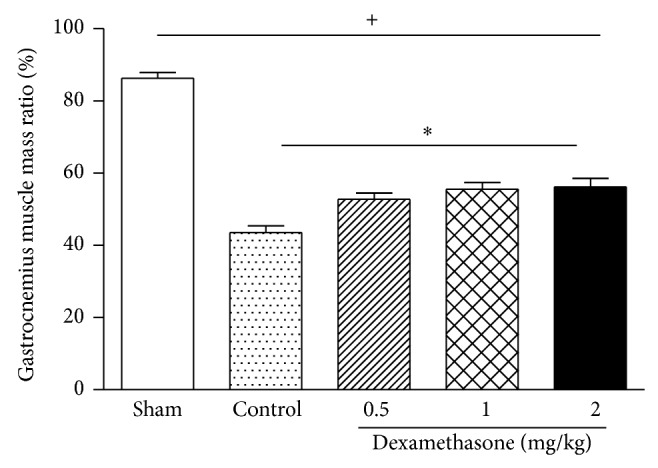
Effects of dexamethasone on the gastrocnemius muscle ratio 28 days after surgery. Rats were administered local intramuscular injections with 0.5, 1, or 2 mg/kg dexamethasone, or saline once daily after surgery. The values are represented as means ± SD for 6 rats per group. ^+^Significant difference compared to sham, *P* < 0.01. ^*^Significant difference compared to control, *P* < 0.05.

As shown in Figure 2, the gastrocnemius muscle ratio between the operated and nonoperated sides in the sham group on day 28 after surgery was 86.40 ± 5.94%. However, in the control group, the gastrocnemius muscle ratio showed a marked decrease to 43.58 ± 5.28%. This decrease was attenuated by treatment with low, moderate, and high doses of dexamethasone (52.76 ± 3.66%, 55.57 ± 6.21%, and 56.17 ± 2.35%, resp.).

### 3.3. Immunohistochemical Staining

**Figure 3:**
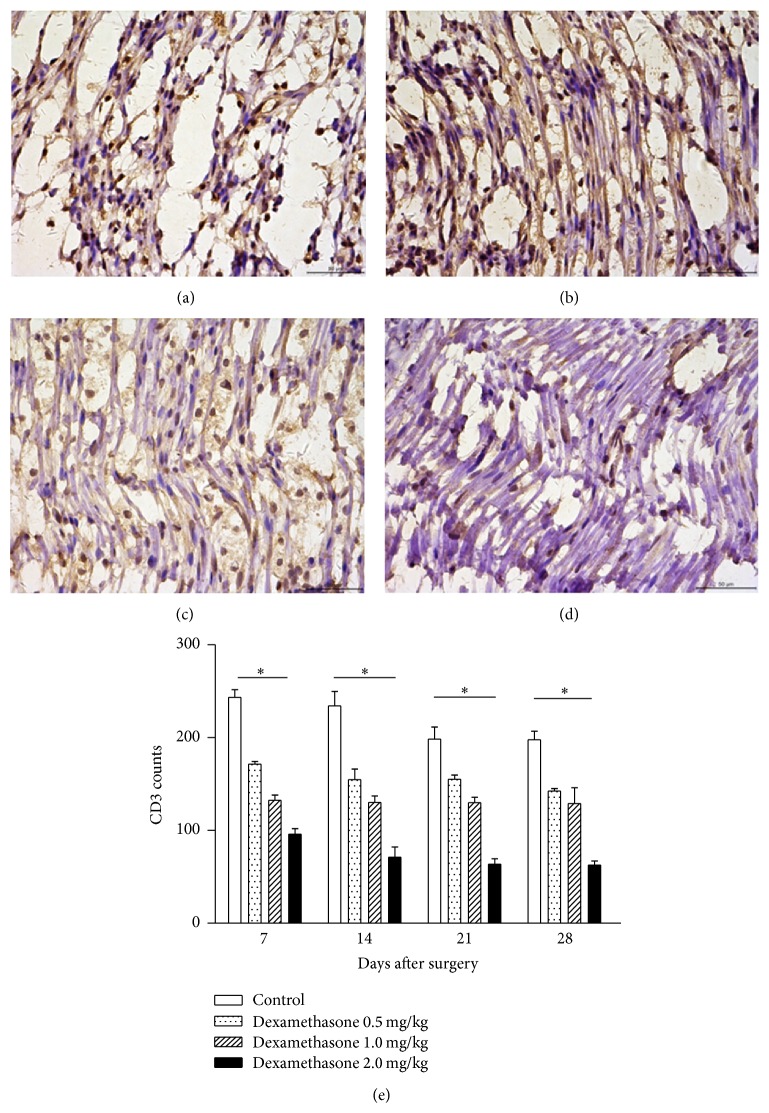
Effects of dexamethasone on T-cell infiltration. ((a)–(d)) Immunohistochemical staining of nerve tissue on day 28 after surgery (magnification, 400x). (a) Control group; ((b)–(d)) 0.5, 1, and 2 mg/kg dexamethasone-treated groups, respectively. Data in the sham group are not shown; (e) CD3-positive cell counts at each time point after surgery. The values represent means ± SD for 6 rats per group. ^*^Significant difference compared to control, *P* < 0.01.

At different time points after surgery, large numbers of CD3-positive cells were found in each group (Figure 3(e)), demonstrating that sciatic nerve injury induced a T-cell recruitment response. Recruitment of CD3-positive cells was assessed on days 7, 14, 21, and 28 after surgery. The control group showed massive T-cell infiltration (197.5 ± 9.47 CD3-positive cells) around the injured region (Figure 3(a)). Administration of dexamethasone at various doses significantly reduced T-cell infiltration, especially at the highest dose (62.5 ± 4.52 CD3-positive cells) (Figures 3(b)–3(d)).

**Figure 4:**
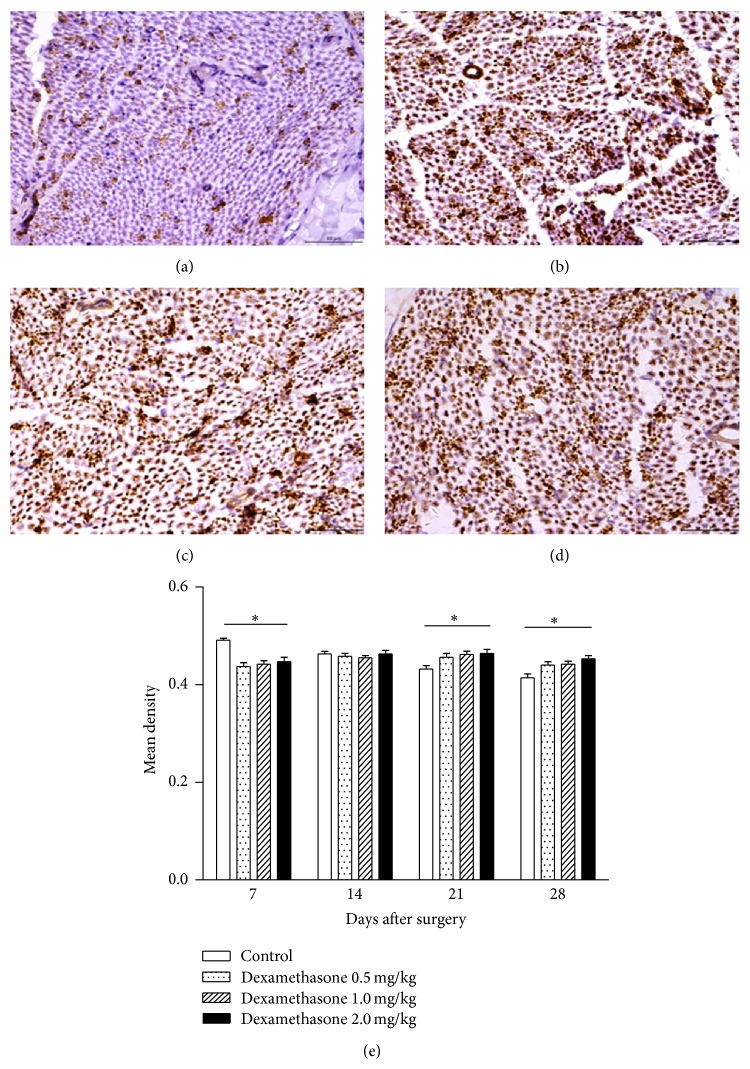
Effects of dexamethasone on mean density of GAP-43. ((a)–(d)) Immunohistochemical staining of nerve tissue on day 28 after surgery (magnification, 400x). (a) Control group; ((b)–(d)) 0.5, 1, and 2 mg/kg dexamethasone-treated groups, respectively. Data in the sham group are not shown. (e) Mean density of GAP-43 at each time point after surgery. The values represent means ± SD for 6 rats per group. ^*^Significant difference compared to control, *P* < 0.05.

GAP-43 expression intensity was also measured at various time points after surgery. The mean density of GAP-43 was highest (0.491 ± 0.004) in control group 7 days after surgery compared to the other three groups. Moreover, its expression gradually decreased in the control group over time. However, in groups (b), (c), and (d), the mean density of GAP-43 was still at a high level on days 21 and 28 after surgery, which was significantly different than control group (a) (*P* < 0.05) (Figure 4). This indicates that administration of dexamethasone enhanced GAP-43 expression levels.

### 3.4. HE Staining

**Figure 5:**
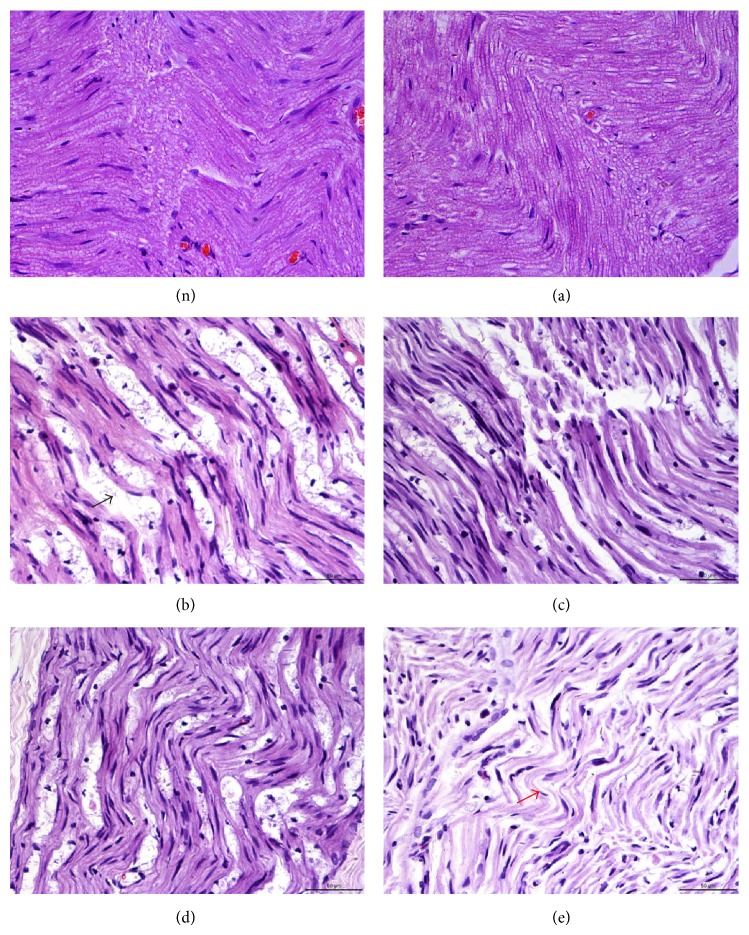
Histological analysis by H&E staining of rat sciatic nerves on day 28 after surgery. (n) Sciatic nerve on nonoperated side. Group (a) (sham) shows well-organized myelin sheets, round axons, and absence of infiltrating cells. Group (b) shows several areas of edema and degraded myelin sheets (black arrow) and several infiltrating mononuclear cells. Groups (c)–(e) show effects of dexamethasone on the injured sciatic nerves. The red arrow shows that alleviated areas of edema and nerve fibers seem to be better organized than those in group (b). Magnification, ×400.

No histopathological changes were observed in group (a) (Figures 5(n) and 5(a)). Medullated fibers appeared to be arranged well and there was no inflammatory cell infiltration. In group (b), crush injury produced severe changes in the sciatic nerve, including swelling of the myelin sheet, vacuolization, and myelin ellipsoids (Figure 5(b)). However, local intramuscular injection with 0.5, 1, and 2 mg/kg dexamethasone alleviated all the histological changes from the crush injury in the sciatic nerve (Figures 5(c), 5(d), and 5(e)).

### 3.5. Neuromorphometry

**Figure 6:**
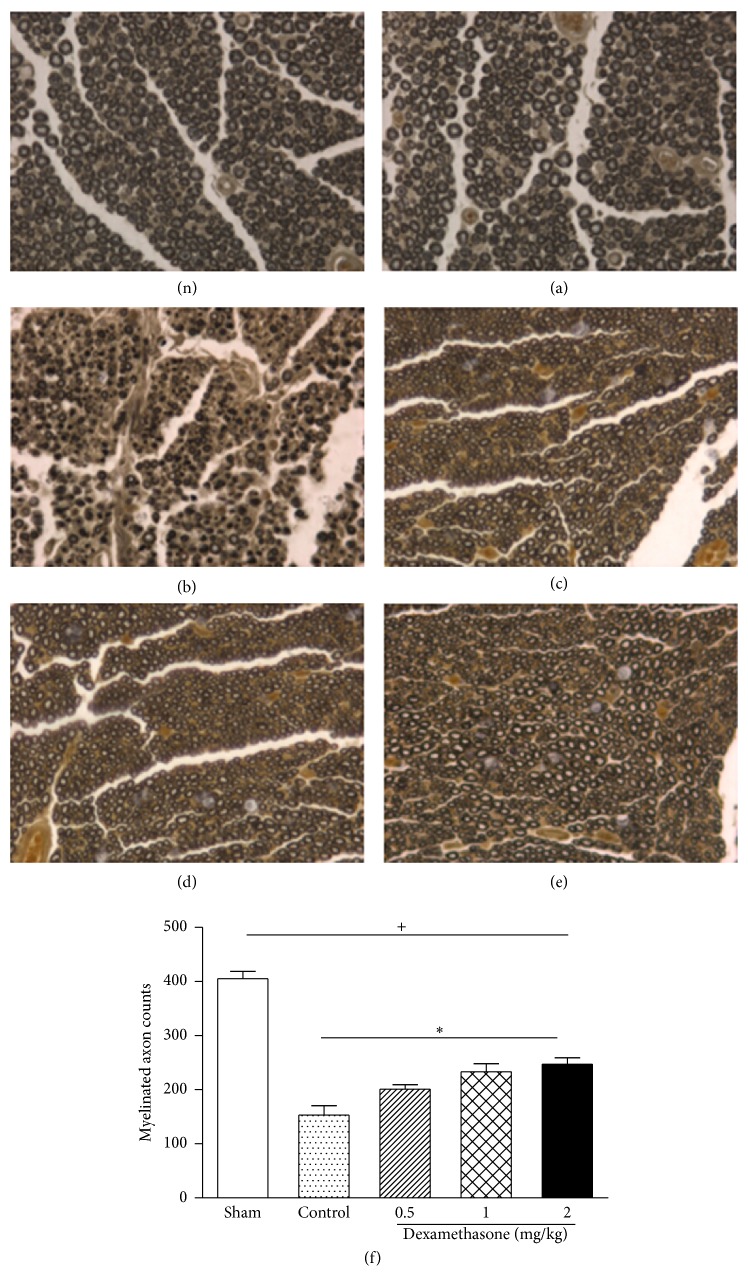
Effects of dexamethasone on the myelinated axon counts on day 28 after surgery. ((n), (a)–(e)) Histological micrographs of nerve tissue stained with osmium tetroxide staining (magnification 400x). (n) shows the sciatic nerve on nonoperated side; (a) sham group; (b) control group; ((c)–(e)) 0.5, 1, and 2 mg/kg dexamethasone-treated groups, respectively. (f) Total myelinated axon counts. Rats were administered local intramuscular injections with 0.5, 1, or 2 mg/kg dexamethasone, or saline once a day after surgery. The values represent means ± SD for 6 rats per group. ^+^Significant difference compared to sham, *P* < 0.01. ^*^Significant difference compared to control, *P* < 0.05.

Staining of cross sections of rat sciatic nerve with osmium tetroxide is a very efficient method for measuring the myelinated nerve fiber area as well as counting myelinated nerve fibers. On day 28 after surgery, the sectioned nerves from each group were stained with osmium tetroxide. The total myelinated axon number per transverse section in the sham group was 425.00 ± 42.3. However, in the control group, myelinated axon numbers showed a significant decrease to 76.8 ± 8.81. This decrease was attenuated by local intramuscular injections of dexamethasone (Figure 6).

## 4. Discussion

After peripheral nerve injury, axonal regeneration does not always allow for adequate functional recovery, and therefore the patients do not recover normal motor control and fine sensibility [13]. In clinical practice, surgical repair is usually required for long distance defect injuries, while drug therapy is a potential choice for treating nerve crush injuries. New and effective drugs that promote nerve regeneration have become an urgent therapeutic need. Dexamethasone is an anti-inflammatory glucocorticoid often used after injury to reduce edema in neurologic tissue and otherwise mitigate the consequences of neural inflammation. However, the pathophysiologic mechanisms of anti-inflammatory agents on the nerve are largely unknown. In this study, we analyzed the effectiveness of local intramuscular injection of dexamethasone on sciatic nerve regeneration using a crush model. Our results showed that application of dexamethasone enhanced functional recovery after sciatic nerve crush injury during the study period.

The rat sciatic nerve model is a widely used model for simultaneous evaluation of motor and sensory nerve function. The walking track analysis clearly demonstrated a direct relationship between individual hind limb muscle function and print measurement. Analysis of rat walking tracts by SFI has proven to be a reliable, repeatable, economical, and quantitative method of evaluating function following sciatic nerve injury and repair [14]. The SFI is considered an assessment of overall nerve function in the rat because walking requires complex motor-unit reinnervation coordinated by cortically integrated sensory feedback. Our data showed that on day 14 after surgery function was still poor in both the control and dexamethasone-administered groups. However, on day 28, the ability to walk began to improve. Better and faster functional recovery was shown in the dexamethasone-administered group, indicating that dexamethasone can promote functional recovery after sciatic nerve crush injury.

Denervation of a target muscle occurs as a consequence of peripheral nerve injury, accompanied by a series of histological and biochemical alterations, leading to final muscle atrophy. If the muscle is reinnervated, then muscle function will be restored and atrophy will stop. The sciatic nerve crush, with the continuity of the nerve being preserved, allows for axonal reinnervation and restoration of the nerve-muscle interaction [15]. The gastrocnemius muscle is supplied by the posterior tibial branch of the sciatic nerve. However, compared with the control group, groups treated with dexamethasone showed accelerated improvement of gastrocnemius muscle atrophy induced by sciatic denervation. These data correlate with our findings of SFIs and suggest that it may be due to appropriate reinnervation.

Recent research has shown that the innate immune system participates in degeneration and regeneration of the peripheral nervous system after injury through molecular inflammatory mediators [16, 17]. One physiological process that explicitly shows the relation between the immune and nervous systems is the regulation of peripheral immune cell infiltration and activation in injured tissue. Peripheral lesions cause nerve damage, production of inflammatory agents, and Schwann cell activation, which lead to subsequent neutrophil, macrophage, and lymphocyte recruitment. Our results confirmed the presence of CD3-positive cells, which indicates T-cell recruitment and activation after sciatic nerve crush injury as a key postlesion event. Furthermore, we demonstrate that treatment with dexamethasone led to a decrease of CD3-positive cells, which mainly contributed to its immunosuppressive effect. Therefore, primary immunosuppression may at least contribute to the neuroprotection by restricting CD3-positive cell invasion.

GAP-43 is associated with the development and plasticity of the nervous system. The protein is maintained at steady levels after the mature synapse contact is completed. During nerve development or injury, the expression of GAP-43 varies over 100-fold from very low levels under resting conditions to high levels. The elevated expression of GAP-43 promotes neurite outgrowth. GAP-43 expression is closely related with periods of active growth cone function and its levels are dramatically increased within regenerating nerves after axonal injury [18, 19]. In the present study, we found that dexamethasone treatment effectively elevated the expression of GAP-43 in the crushed nerve on days 21 and 28 after surgery as compared to saline treatment. Based on our results, we postulate that dexamethasone promotes the regeneration and functional recovery of the injured sciatic nerves through upregulation of GAP-43 expression. Nevertheless, further studies are necessary to elucidate the mechanism by which dexamethasone could upregulate GAP-43 expression in peripheral nerves.

We also employed several morphological analyses on rat sciatic nerves subjected to injury and treatment by dexamethasone. H&E staining of sciatic nerves strongly supported the beneficial effect of dexamethasone in axonal regeneration due to reduced edema area as well as fewer degraded myelin sheets in dexamethasone-administered groups compared to the control group. Previous studies have indicated that dexamethasone not only reduces the extent of demyelination but also enhances remyelination. Our osmium tetroxide staining results showed greater numbers of myelinated axons in dexamethasone-administration groups. These results suggest that treatment with dexamethasone promotes functional recovery by accelerating axonal regeneration after sciatic nerve crush injury in the rat.

In conclusion, in this study we demonstrated that dexamethasone promotes peripheral nerve repair in a rat model of sciatic nerve injury through the inhibition of CD3-positive cell infiltration as well as the upregulation of GAP-43 expression. These findings provide new insight into the neuroprotective and neurotrophic effects of dexamethasone and support the application of these agents in clinical treatment of peripheral nerve injury.

## References

[1] Kim T.-H., Yoon S.-J., Lee W.-C. (2011). Protective effect of GCSB-5, an herbal preparation, against peripheral nerve injury in rats. *Journal of Ethnopharmacology*.

[2] Burnett M. G., Zager E. L. (2004). Pathophysiology of peripheral nerve injury: a brief review. *Neurosurg Focus*.

[3] Steinman L. (2004). Elaborate interactions between the immune and nervous systems. *Nature Immunology*.

[4] Watkins L. R., Hutchinson M. R., Milligan E. D., Maier S. F. (2007). ‘Listening’ and ‘talking’ to neurons: implications of immune activation for pain control and increasing the efficacy of opioids. *Brain Research Reviews*.

[5] Salegio E. A. A., Pollard A. N., Smith M., Zhou X.-F. (2010). Sciatic nerve conditioning lesion increases macrophage response but it does not promote the regeneration of injured optic nerves. *Brain Research*.

[6] Xavier A. M., Serafim K. G. G., Higashi D. T. (2012). Simvastatin improves morphological and functional recovery of sciatic nerve injury in Wistar rats. *Injury*.

[7] Koeppen A. H. (2004). Wallerian degeneration: history and clinical significance. *Journal of the Neurological Sciences*.

[8] Zochodne D. W. (2000). The microenvironment of injured and regenerating peripheral nerves. *Muscle & Nerve Supplements*.

[9] Sun H., Yang T., Li Q. (2012). Dexamethasone and vitamin B12 synergistically promote peripheral nerve regeneration in rats by upregulating the expression of brain-derived neurotrophic factor. *Archives of Medical Science*.

[10] Mohammadi R., Azad-Tirgan M., Amini K. (2013). Dexamethasone topically accelerates peripheral nerve repair and target organ reinnervation: a transected sciatic nerve model in rat. *Injury*.

[11] Bain J. R., Mackinnon S. E., Hunter D. A. (1989). Functional evaluation of complete sciatic, peroneal, and posterior tibial nerve lesions in the rat. *Plastic and Reconstructive Surgery*.

[12] Canan S., Bozkurt H. H., Acar M. (2008). An efficient stereological sampling approach for quantitative assessment of nerve regeneration. *Neuropathology and Applied Neurobiology*.

[13] Allodi I., Udina E., Navarro X. (2012). Specificity of peripheral nerve regeneration: interactions at the axon level. *Progress in Neurobiology*.

[14] Dinh P., Hazel A., Palispis W., Suryadevara S., Gupta R. (2009). Functional assessment after sciatic nerve injury in a rat model. *Microsurgery*.

[15] Yuan Y., Shen H., Yao J., Hu N., Ding F., Gu X. (2010). The protective effects of Achyranthes bidentata polypeptides in an experimental model of mouse sciatic nerve crush injury. *Brain Research Bulletin*.

[16] Ha G. K., Parikh S., Huang Z., Petitto J. M. (2008). Influence of injury severity on the rate and magnitude of the T lymphocyte and neuronal response to facial nerve axotomy. *Journal of Neuroimmunology*.

[17] Temporin K., Tanaka H., Kuroda Y. (2008). Interleukin-1 beta promotes sensory nerve regeneration after sciatic nerve injury. *Neuroscience Letters*.

[18] Irwin N., Chao S., Goritchenko L. (2002). Nerve growth factor controls GAP-43 mRNA stability via the phosphoprotein ARPP-19. *Proceedings of the National Academy of Sciences of the United States of America*.

[19] Benowitz L. I., Routtenberg A. (1997). GAP-43: an intrinsic determinant of neuronal development and plasticity. *Trends in Neurosciences*.

